# Mobile App–Based Intervention and Cardiovascular Risk Factors in Patients With Uncontrolled Type 2 Diabetes: A Randomized Clinical Trial

**DOI:** 10.1001/jamanetworkopen.2025.29762

**Published:** 2025-09-02

**Authors:** Pei-Zhen Zhang, Dan Guo, Chang-Qin Liu, Ning Chen, Jian-Fang Liu, Xu-Zhen Lei, Lin-Jie Yang, Ya-Ting Liu, Xu Li, Jun-Feng Huang, Chun-Min Du, Kai Wang, Wei Mo, Jia-Yang Lin, Chen-Si-Han Huang, Bing-Yan Xu, Xue-Yun Wei, De-Ying Liu, Jun-Lin Huang, Yan Huang, Yao-Ming Xue, Yan-Mei Zeng, Shi-Qun Liu, Zhi-Min Ma, Hui-Jie Zhang

**Affiliations:** 1 Department of Endocrinology and Metabolism, Nanfang Hospital, Southern Medical University, Guangzhou, China; 2 Department of Endocrinology and Diabetes, The First Affiliated Hospital of Xiamen University, School of Medicine, Xiamen University, Xiamen, China; 3 Department of Endocrinology, Fudan University Zhongshan Hospital Xiamen Branch, Xiamen, China; 4 Department of Endocrinology & Metabolism, Guangdong Second Traditional Chinese Medicine Hospital, Guangzhou, China; 5 Department of Endocrinology, Suzhou Research Center of Medical School, Suzhou Hospital, Affiliated Hospital of Medical School, Nanjing University, Suzhou, China; 6 Guangdong Provincial Key Laboratory of Shock and Microcirculation, Guangzhou, China; 7 State Key Laboratory of Organ Failure Research, Guangzhou, China

## Abstract

**Importance:**

Controlling modifiable cardiovascular risk factors is important but underused for patients with type 2 diabetes (T2D). Mobile message–based intervention strategies could address this gap but lack evidence of benefit on multiple risk factors.

**Objective:**

To evaluate the effectiveness of a mobile message–based intervention in controlling cardiovascular risk factors in patients with T2D.

**Design, Setting, and Participants:**

In this randomized clinical trial, adults with uncontrolled T2D comorbid with cardiovascular disease (CVD) risk factors were recruited from 5 clinical centers in China. Data were collected from November 2018 to March 2022 and analyzed from January to June 2023.

**Intervention:**

Participants were randomized to receive either usual care or a mobile message–based intervention of 6 text messages per week from different modules designed to remind, encourage, and motivate them to participate in the behaviors needed for improving glycemic control and CVD risk factor management for 12 months.

**Main Outcomes and Measures:**

The primary outcome included mean changes in hemoglobin A_1c_ (HbA_1c_), low-density lipoprotein cholesterol (LDL-C), and systolic blood pressure (SBP) levels across 12 months. The secondary outcomes included the percentage of participants with controlled HbA_1c_ at 12 months. Data were analyzed using the intention-to-treat principle.

**Results:**

A total of 819 participants (552 men [67.4%]; mean [SD] age, 50.1 [11.9] years; mean [SD] HbA_1c_ level, 10.2% [2.1%]) were enrolled, of whom 410 were randomized to the intervention group and 409 to the control group. During the 12-month intervention, significant reductions in the mobile message–based intervention group were observed for HbA_1c _levels by −2.8% (95% CI, −2.9% to −2.6%), LDL-C by −11.1 mg/dL (95% CI, −14.7 to −7.4 mg/dL), and SBP by −2.5 mm Hg (95% CI, −3.9 to −1.2 mm Hg), and in the usual care group, by −2.5% (95% CI, −2.7% to −2.3%) for HbA_1c_, −11.9 mg/dL (95% CI, −15.8 to −8.0 mg/dL) for LDL-C, and −0.1 mm Hg (95% CI, −1.6 to 1.3 mm Hg) for SBP. The net group differences were −0.3% (95% CI, −0.5% to −0.0%) for HbA_1c_, 0.9 mg/dL (95% CI, −4.5 to 6.2 mg/dL) for LDL-C, and −2.4 mm Hg (95% CI, −4.3 to −0.4 mm Hg) for SBP (*P* = .001 for the combined overall effect). The percentage of participants with controlled HbA_1c_ among all participants was significantly higher in the intervention group than in the control group at 12 months (195 participants [54.0%] vs 146 participants [46.1%]; *P* = .04).

**Conclusions and Relevance:**

In this randomized clinical trial of adults with uncontrolled T2D in China, a mobile message–based intervention resulted in a modest improvement in HbA_1c_ and SBP in patients with diabetes compared with usual care. These results suggest that mobile message–based strategies for improving glycemic control and CVD risk factors should be considered for adults with T2D.

**Trial Registration:**

ClinicalTrials.gov Identifier: NCT03724526

## Key Points

**Question:**

Can a mobile message–based intervention improve cardiovascular risk factors among patients with uncontrolled type 2 diabetes?

**Findings:**

In this 12-month randomized clinical trial of 819 adults with uncontrolled type 2 diabetes comorbid with cardiovascular disease risk factors, when compared with usual care, adults receiving the message-based intervention had significant improvements in hemoglobin A_1c_ levels and systolic blood pressure.

**Meaning:**

These findings suggest that mobile message–based strategies for improving glycemic control and cardiovascular disease risk factors should be considered for adults with type 2 diabetes.

## Teaser

This randomized clinical trial compares the effectiveness across 12 months of usual care with a mobile message–based intervention designed to remind, encourage, and motivate adults with type 2 diabetes and cardiovascular risk factors to participate in behaviors needed for improving glycemic control and risk factor management.

## Introduction

The growing prevalence of type 2 diabetes (T2D) and the consequent cardiovascular disease (CVD) pose a major global health issue. It is estimated that 537 million people had diabetes in 2021, and this number is projected to reach 783 million by 2045.^1^ In China, the prevalence of diabetes in adults aged 20 years or older increased to 12.4% in 2018,^2^ and 86% of Chinese patients with diabetes have multiple comorbid conditions, such as obesity, hypertension, dyslipidemia, and CVD.^3^ Clinical evidence has shown that controlling multiple cardiovascular risk factors lowers the incidence of CVD.^4,5,6^ However, such control remains a clinical challenge as shown by the relatively low proportion of patients with diabetes who have controlled CVD risk factors.^7^ In China, only 5.6% of adults with diabetes have simultaneously achieved therapeutic targets for hemoglobin A_1c_ (HbA_1c_), blood pressure (BP), and serum cholesterol.^8^

Barriers for diabetes management, such as lack of health knowledge and motivation to control risk factors, poor adherence to medication therapy, lack of social support, difficulty communicating with physicians, and insufficient time and resources of the health care professional, contribute to poor control of glycemic and CVD risk factors.^9,10^ Mobile health, which is commonly used as a health care delivery strategy to promote health, can address barriers and facilitate disease self-management by directly delivering health information and electronic reminders to patients.^11,12,13^ Accumulating evidence has shown that mobile message–based interventions improve medication adherence, lifestyle modification, and glycemic control among patients with diabetes.^14,15,16,17,18,19,20^ However, most existing studies have relatively small sample sizes, short intervention times (ie, 3-9 months), and different patterns of text messaging interventions. Importantly, these existing clinical trials of mobile message–based interventions conducted in populations with diabetes are also focused only on glycemic control rather than on control of multiple CVD risk factors. Indeed, most patients with diabetes require long-term multifactorial therapy to simultaneously reduce multiple risk factors because of their multiple comorbid conditions.^4,6^ Therefore, evaluating the effects of mobile message–based interventions on reduction of CVD risk factors among patients with diabetes is needed to fill the knowledge gaps. In this randomized clinical trial, we evaluated the effectiveness of a mobile message–based intervention on CVD risk factor management for patients with uncontrolled T2D.

## Methods

### Study Design

This was a multicenter, parallel randomized clinical trial conducted in 5 clinic centers in China. Eligible participants with uncontrolled T2D were randomly assigned to a mobile message–based intervention or to usual care for 12 months. The primary outcome included the mean changes in HbA_1c_ levels, low-density lipoprotein cholesterol (LDL-C) levels, and systolic blood pressure (SBP) levels from baseline to 12 months simultaneously modeled for a single overall treatment effect. The trial protocol is provided in Supplement 1 and was approved by the ethics committees of Nanfang Hospital of Southern Medical University and the other clinical centers. The trial was conducted in accordance with the principles of the Declaration of Helsinki.^21^ All participants provided written informed consent. This report adhered to the Consolidated Standards of Reporting Trials (CONSORT) reporting guideline.

### Participants

We recruited all the participants during outpatient visits or hospitalization from 5 clinic centers in China between October 2018 and February 2021. Participants were eligible if they were 18 years of age or older with uncontrolled T2D (defined as HbA_1c_ ≥7.0%, or ≥7.5% if combined with clinical CVD [to convert to proportion of total hemoglobin, multiply by 0.01]) comorbid with at least 1 risk factor for CVD (SBP ≥140 mm Hg or diastolic BP ≥90 mm Hg or LDL-C ≥100 mg/dL [to convert to millimoles per liter, multiply by 0.0259]) or clinically diagnosed atherosclerotic CVD (including acute coronary syndrome, ischemic stroke, transient ischemic attack, or peripheral artery disease). All participants had access to a smartphone to read and accept text messages. Exclusion criteria included serious heart failure (New York Heart Association class III to IV), end-stage kidney disease, current or planned pregnancy, and inability to adhere to a 12-month follow-up.

### Randomization

Eligible participants were randomly assigned to either the mobile message–based intervention or to the control group in a 1:1 ratio. Randomization was stratified by study centers and performed centrally at the Research and Data Coordinating Center in Nanfang Hospital. The randomization schedules were generated using the PROC PLAN procedure in SAS statistical software version 9.4 (SAS Institute) with a block size of 6. Owing to the nature of the intervention, participants were inevitably aware of their group assignments. The research nurses, investigators, and statisticians were blinded to the treatment allocation. The clinical outcomes were also collected by research physicians blinded to the treatment during the study period.

### Intervention

The mobile message–based intervention was mobile text messages that were regularly delivered by the SMS (short messaging service) platform to remind, encourage, and motivate patients to participate in the behaviors needed for improving glycemic control and CVD risk factor management. Based on recommendations from the Chinese Diabetes Society^22^ and the American Diabetes Association,^23^ a text message bank in plain language was established and modified for Chinese diet, culture, and language habits (text message examples and more details in Supplement 1). All messages were in text-compatible 70-character phrases in Chinese. The text message bank covered the following 7 topics: general information about CVD and T2D, healthy diet, physical activity, medication adherence, glucose monitoring, BP control, and smoking cessation. The bank was regularly updated based on newly published guidelines to ensure diversity.

Participants assigned to the mobile message–based intervention group received 6 text messages per week from different modules and usual care during the 12-month study period; the control group received usual care. Researchers saved and checked the delivery log to ensure successful delivery. At each visit, participants in the intervention group were asked to confirm or update their phone number and complete a dedicated satisfaction survey questionnaire to investigate the proportion read and satisfaction with the text messages.

### Outcomes and Data Collection

Data were collected from November 2018 to March 2022. The primary outcome comprised the overall mean changes in HbA_1c_, LDL-C, and SBP levels from baseline to 12 months simultaneously modeled for a single overall treatment effect. Secondary outcomes included the proportion of participants with controlled HbA_1c_ (<7.0%, or <7.5% for patients with CVD), LDL-C (<100 mg/dL), and BP (<140/90 mm Hg) levels at 12 months, the net change in fasting blood glucose (FBG) levels, total cholesterol levels, triglyceride levels, high-density lipoprotein cholesterol levels, diastolic BP levels, body mass index (BMI, calculated as weight in kilograms divided by height in meters squared), self-reported medication adherence, and health-related quality of life. Medication adherence was measured by the Morisky Medication Adherence Scale (MMAS) (range, 0 to 8, with lower scores indicating lower adherence). Quality of life was measured with the 12-item Short-Form Health Survey Questionnaire. Lifestyle behaviors, including physical activity and daily calorie intake, were also assessed in the study using the International Physical Activity Questionnaire^24^ and a standardized diet questionnaire, respectively.

Information on sociodemographic factors (age, sex, educational level, and employment status), lifestyle habits (smoking status, drinking status, physical activity, and diet), medication, and medical history was collected by trained staff. Data on physical measurements (height, weight, waist circumference, and BP) were collected at baseline and follow-up visits every 3 months for all participants by trained researchers with the use of standard methods. Measurements of height, weight, and waist circumference were performed in sets of 2 replicates. Sitting BP levels were measured in triplicate after 5 minutes of quiet rest using an automatic electronic sphygmomanometer (Omron). The mean value was used for analysis. Biochemical laboratory tests, including levels of HbA_1c_, FBG, and LDL-C, were measured at the central laboratory according to the manufacturer’s instructions using 10-hour overnight fasting blood samples at 3-month intervals. Participants in the intervention group were asked to complete a short questionnaire at follow-up about the satisfaction and acceptability of the text messaging intervention. Adverse events were queried during clinical follow-up visits through face-to-face investigation and participant self-report. Notably, due to the impact of the COVID-19 pandemic, the original 2-week follow-up window was extended to 4 weeks during peak pandemic restrictions (January to May 2020).

### Statistical Analysis

We estimated that a sample size of 800 participants would provide 80% power to detect a 5% difference of the net change in standardized overall treatment effect at a significance level of .05 using a 2-tailed test. This result corresponded to a detectable difference of 0.5% in HbA_1c_ change (7.2% ± 1.6% at baseline), 5 mm Hg in SBP (132 ± 18 mm Hg at baseline) and 9.7 mg/dL in LDL-C (113.5 ± 37.1 mg/dL at baseline).^15,16,25^ This sample size allowed for a dropout rate of 20% across 12 months.

Data were analyzed according to the intention-to-treat principle. Continuous variables are presented as means (SDs) or medians (IQRs), and categorical variables are presented as frequencies with percentages. Group differences in the trial outcomes were evaluated with the use of general linear models for continuous variables and the χ^2^ test for categorical variables. The overall intervention effect on HbA_1c_, LDL-C, and SBP levels was tested using a scaled marginal model in which each outcome was scaled by its SD. Generalized estimating equations were used to calculate the regression coefficient and outcome-specific scale variables in SAS PROC GENMOD, and the assumption of an overall intervention effect was tested using a score test.^26^ Group differences for the secondary outcomes were also evaluated with the use of generalized estimating equations for continuous variables, adjusting for baseline levels corresponding to each study outcome. In addition, the correlations of outcomes among participants within each clinic center using an exchangeable correlation structure were taken into account in the analysis. Missing data were handled by multiple imputations for a random missingness pattern with the use of the Markov Chain Monte Carlo method.

Subgroup analyses of HbA_1c_, LDL-C, and SBP levels were conducted by age (<65 or ≥65 years), sex (male or female), educational level (junior high school or below, high school or above), employment status (never worked or unemployed, retired, and employed), smoking status (never, former, or current), current alcohol consumption (yes or no), prevalent obesity (yes [BMI ≥28] or no [BMI <28]), prevalent hypertension (yes or no), and prevalent hypercholesterolemia (yes or no). Analyses were conducted from January to June 2023 using SAS version 9.4 (SAS Institute). A 2-sided *P* < .05 was considered to indicate statistical significance for the outcomes.

## Results

**Figure 1.**
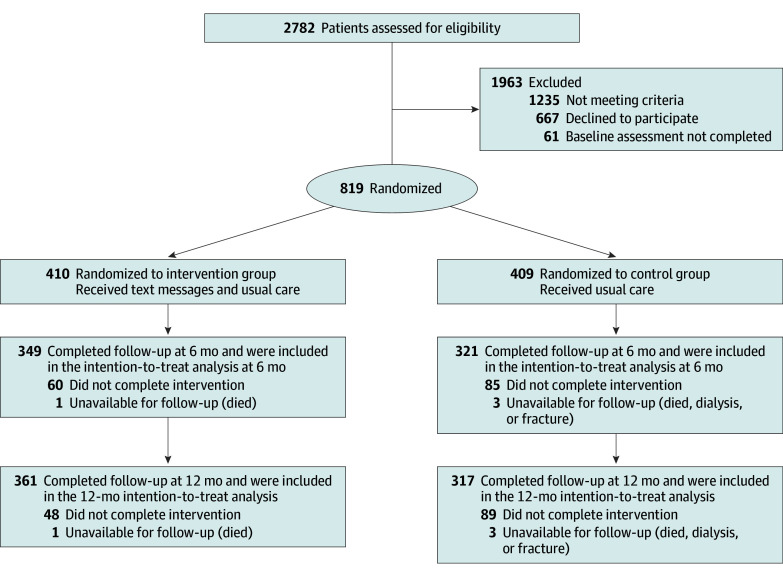
Flowchart of Trial Participants

Between October 2018 and February 2021, a total of 2782 patients with diagnosed T2D were screened for eligibility. Of these, 819 participants were enrolled from 5 clinic centers and randomly assigned to the intervention (n = 410) or control group (n = 409) (Figure 1). In total, 361 participants (88.0%) in the intervention group and 317 participants (77.5%) in the control group completed the 12-month intervention. Their mean (SD) age was 50.1 (11.9) years; 552 (67.4%) were male and 267 (32.6%) were female. Their mean (SD) HbA_1c_ level was 10.2% (2.1%), mean (SD) SBP was 126.9 (14.0) mm Hg, and mean (SD) LDL-C level was 129.8 (34.5) mg/dL. Baseline characteristics of the study participants were similar between the 2 groups (Table 1).

**Table 1.** Characteristics of the Participants at Baseline

Characteristic	Participants, No. (%)^a^	
	Intervention group (n = 410)	Control group (n = 409)
Sex		
Female	125 (30.5)	142 (34.7)
Male	285 (69.5)	267 (65.3)
Age, mean (SD), y	49.4 (12.2)	50.8 (11.6)
Marital status		
Single	19 (4.6)	16 (3.9)
Married or cohabited	379 (92.4)	376 (91.9)
Separated	6 (1.5)	7 (1.7)
Widowed	6 (1.5)	10 (2.4)
Highest formal educational level		
None	7 (1.7)	12 (2.9)
Primary school	61 (14.9)	64 (15.6)
Junior high school	102 (24.9)	133 (32.5)
Senior high school or technical college	115 (28.1)	107 (26.2)
College or university undergraduate	118 (28.8)	86 (21.0)
University postgraduate	7 (1.7)	7 (1.7)
Employment status		
Never worked or unemployed	41 (10.0)	55 (13.4)
Retired	118 (28.8)	112 (27.4)
Employed	251 (61.2)	242 (59.2)
Smoking status		
Never	215 (52.4)	229 (56.0)
Former	68 (16.6)	68 (16.6)
Current	127 (31.0)	112 (27.4)
Current alcohol consumption	69 (16.8)	67 (16.4)
Body weight, mean (SD), kg	68.9 (11.7)	67.6 (12.6)
BMI, mean (SD)	25.2 (3.6)	25.0 (3.7)
Waist circumference, mean (SD), cm	89.8 (9.0)	89.2 (10.0)
Systolic blood pressure, mean (SD), mm Hg	127.5 (14.5)	126.2 (13.4)
Diastolic blood pressure, mean (SD), mm Hg	78.9 (9.7)	77.8 (8.3)
Fasting glucose, mean (SD), mg/dL	176.2 (88.5)	176.4 (90.5)
Hemoglobin A_1c_, mean (SD), %	10.1 (2.0)	10.3 (2.2)
Total cholesterol, mean (SD), mg/dL	202.2 (46.6)	202.0 (45.0)
Triglyceride, median (IQR), mg/dL	143.8 (100.0-220.4)	140.7 (98.2-207.1)
LDL-C, mean (SD), mg/dL	129.7 (34.9)	129.8 (34.1)
HDL-C, mean (SD), mg/dL	42.6 (10.7)	43.8 (12.3)
SF-12, mean (SD), score^b^		
Physical component summary	42.0 (5.8)	41.5 (6.8)
Mental component summary	47.0 (5.0)	47.2 (5.5)
Patient Health Questionnaire-9, median (IQR), score^c^	3 (0-6)	3 (1-6)
History of hypertension	142 (34.6)	139 (34.0)
History of CVD	148 (36.1)	139 (34.0)
History of dyslipidemia	272 (66.3)	266 (65.0)
Medication		
Antihyperglycemic agent	259 (63.2)	273 (66.7)
Antihypertensive drug	106 (25.9)	99 (24.2)
Lipid-lowering agent	61 (14.9)	71 (17.4)

Abbreviations: BMI, body mass index (calculated as weight in kilograms divided by height in meters squared); CVD, cardiovascular disease; HDL-C, high-density lipoprotein cholesterol; LDL-C, low-density lipoprotein cholesterol; SF-12, 12-item Short-Form Health Survey Questionnaire.

SI conversion factors: To convert cholesterol, fasting blood glucose, and triglyceride levels to millimoles per liter, multiply values by 0.0259, 0.0555, and 0.0113, respectively; hemoglobin A_1c_ to proportion of total hemoglobin, multiply by 0.01.

^a^ Percentages may not total 100 because of rounding.

^b^ The SF-12 measures health-related quality of life and includes a physical composite score and a mental composite score, each ranging from 0 to 100, with higher scores indicating better states of health.

^c^ Patient Health Questionnaire-9 scores ranged from 0 to 27, with higher values indicating greater depression severity.

### Primary Outcomes

The mobile message–based intervention had a significant overall improvement on the mean changes in HbA_1c _(−3.0% [95% CI, −0.5% to −0.0%]), LDL-C (0.9mg/dL [95% CI, −4.5 to 6.2 mg/dL]), and SBP (−2.4mm Hg [95% CI, −4.3 to −0.4mm Hg]) levels at 12 months (*P* < .001 for the combined overall effect). HbA_1c_ levels and LDL-C levels significantly decreased from baseline to 12 months in both groups, whereas SBP levels did not (Table 2). Significant reductions in the mobile message–based intervention group were observed for HbA_1c_ levels by −2.8% (95% CI, −2.9% to −2.6%), LDL-C by −11.1 mg/dL (95% CI, −14.7 to −7.4 mg/dL), and SBP by −2.5 mm Hg (95% CI, −3.9 to −1.2 mm Hg), and in the usual care group by −2.5% (95% CI, −2.7% to −2.3%) for HbA_1c_, −11.9 mg/dL (95% CI, −15.8 to −8.0 mg/dL) for LDL-C, and −0.1 mm Hg (95% CI, −1.6 to 1.3 mm Hg) for SBP. The net group differences for the 6-month changes were −0.4% (95% CI, −0.6 to −0.2%) for HbA_1c_, 2.8 mg/dL (95% CI, −2.6 to 8.2 mg/dL) for LDL-C, and −2.3 mm Hg (95% CI, −4.2 to −0.3 mm Hg) for SBP (*P* < .001 for the combined overall effect). In a sensitivity analyses using multiple imputed data, the overall improvements of the mobile message–based intervention on the mean changes in HbA_1c_ levels, LDL-C levels, and SBP levels were similar and significant for the combined overall effect. The group difference in the reduction in HbA_1c_ level was −0.2% (95% CI, −0.5% to −0.0%), in LDL-C level was 0.7 mg/dL (95% CI, −4.7 to 6.2 mg/dL), and in SBP level was −2.3 mm Hg (95% CI, −4.3 to −0.4 mm Hg) across 12 months (eTable 1 in Supplement 2).

**Table 2.** Primary and Secondary End Point Analyses at 6 and 12 Months of Follow-Up Without Imputation for Missing Data

Study outcome	Group, change (95% CI)^a^		Difference between groups, change (95% CI)^b^	*P* value
	Intervention (n = 361)	Control (n = 317)		
**Primary outcomes at 12 mo**				
Change in hemoglobin A_1c_, %	−2.8 (−2.9 to −2.6)	−2.5 (−2.7 to −2.3)	−0.3 (−0.5 to −0.0)	.03
Change in LDL-C, mg/dL	−11.1 (−14.7 to −7.4)	−11.9 (−15.8 to −8.0)	0.9 (−4.5 to 6.2)	.75
Change in systolic BP, mm Hg	−2.5 (−3.9 to −1.2)	−0.1 (−1.6 to 1.3)	−2.4 (−4.3 to −0.4)	.02
**Primary outcomes at 6 mo^c^**				
Change in hemoglobin A_1c_, %	−3.1 (−3.2 to −2.9)	−2.7 (−2.9 to −2.5)	−0.4 (−0.6 to −0.2)	.001
Change in LDL-C, mg/dL	−10.8 (−14.5 to −7.1)	−13.6 (−17.5 to −9.7)	2.8 (−2.6 to 8.2)	.31
Change in systolic BP, mm Hg	−3.2 (−4.5 to −1.8)	−0.9 (−2.3 to 0.5)	−2.3 (−4.2 to −0.3)	.03
**Secondary outcomes**				
Change in fasting plasma glucose, mmol/L				
6 mo	−42.5 (−48.3 to −36.6)	−32.1 (−38.4 to −25.8)	−10.5 (−19.1 to −1.8)	.02
12 mo	−33.0 (−38.4 to −27.4)	−26.8 (−32.8 to −21.1)	−5.9 (−14.1 to 2.2)	.15
Change in total cholesterol, mg/dL				
6 mo	−16.9 (−21.5 to −12.4)	−15.7 (−20.5 to −10.9)	−1.2 (−7.8 to 5.4)	.71
12 mo	−12.9 (−17.3 to −8.4)	−13.6 (−18.4 to −8.8)	0.7 (−5.8 to 7.3)	.82
Change in triglycerides, mg/dL				
6 mo	−28.9 (−48.3 to −9.6)	−9.6 (−29.9 to 10.8)	−19.4 (−47.4 to 8.6)	.18
12 mo	−16.0 (−35.0 to 3.0)	−5.0 (−25.3 to 15.2)	−11.0 (−38.8 to 16.8)	.44
Change in HDL-C, mg/dL				
6 mo	2.9 (2.0 to 3.9)	3.3 (2.4 to 4.3)	−0.4 (−1.8 to 1.0)	.56
12 mo	3.2 (2.2 to 4.1)	3.1 (2.2 to 4.1)	0.0 (−1.3 to 1.4)	.96
Change in diastolic BP, mm Hg				
6 mo	−1.8 (−2.7 to −0.9)	−0.8 (−1.7 to 0.1)	−1.0 (−2.3 to 0.2)	.10
12 mo	−1.4 (−2.3 to −0.6)	−0.6 (−1.5 to 0.3)	−0.9 (−2.1 to 0.4)	.17
Change in BMI				
6 mo	−0.2 (−0.4 to −0.1)	−0.2 (−0.4 to −0.0)	−0.0 (−0.3 to 0.2)	.79
12 mo	−0.1 (−0.2 to 0.1)	−0.0 (−0.2 to 0.1)	−0.0 (−0.3 to 0.2)	.78
Participants with controlled hemoglobin A_1c_ at 12 mo, No. (%)	195 (54.0)	146 (46.1)	NA	.04
Participants with LDL-C <100 mg/dL at 12 mo, No. (%)	118 (32.7)	106 (33.3)	NA	.86
Participants with BP <140/90 mm Hg at 12 mo, No. (%)	294 (80.8)	242 (76.8)	NA	.21

Abbreviations: BMI, body mass index (calculated as weight in kilograms divided by height in meters squared); BP, blood pressure; HDL-C, high-density lipoprotein cholesterol; LDL-C, low-density lipoprotein cholesterol; NA, not applicable.

SI conversion factors: To convert cholesterol, fasting blood glucose, and triglyceride levels to millimoles per liter, multiply values by 0.0259, 0.0555, and 0.0113, respectively; hemoglobin A_1c_ to proportion of total hemoglobin, multiply by 0.01.

^a^ Participant numbers reported here represent those included in the 12-month follow-up analysis.

^b^ The difference in single study outcome between groups was tested using a mixed-effects model in PROC MIXED, with randomized treatment as a factor and baseline end point value or clinic center as a covariate.

^c^ At the 6-month follow-up, the analysis included 349 participants in the intervention group and 321 in the control group.

### Secondary Outcomes

The percentage of participants with controlled HbA_1c_ was significantly higher in the intervention group than in the control group at 12 months (195 participants [54.0%] vs 146 participants [46.1%]; *P* = .04). There were no significant group differences in the percentages of patients with controlled LDL-C (118 [32.7%] vs106 [33.3%]; *P* = .86) and SBP (294 [80.8%] vs 242 [76.8%]; *P* = .21) (Table 2). The FBG levels at the 6-month follow-up were lower in the intervention group compared with the control group, with a mean difference of −10.5 mg/dL (95% CI, −19.1 to 1.8 mg/dL [to convert to millimoles per liter, multiply by 0.0555]; *P* = .02), but the net difference was no longer statistically significant at 12 months (−5.9 mg/dL [95% CI, −14.1 to 2.2 mg/dL]; *P* = .15). There was no significantly greater reduction in waist circumference, weight, BMI, diastolic BP, or lipid levels (ie, total cholesterol, triglyceride, and high-density lipoprotein cholesterol) in the intervention group than in the control group across 12 months (Table 2; eFigures 1 and 2 in Supplement 2).

**Figure 2.**
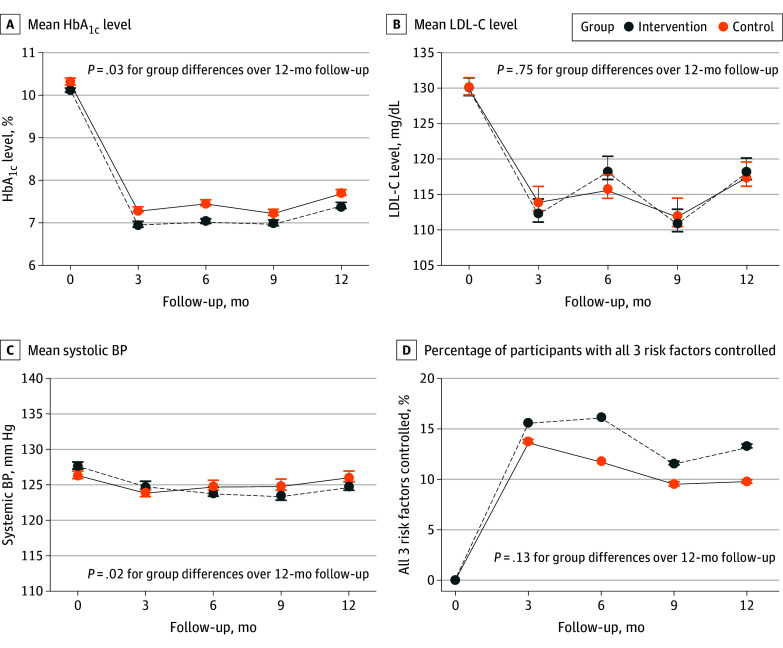
Absolute Hemoglobin A_1c_ (HbA_1c_), Low-Density Lipoprotein Cholesterol (LDL-C), Systolic Blood Pressure (BP) Levels, and Percentages of Participants With All 3 Risk Factors Controlled for Intervention and Control Groups Error bars represent SEs for means. To convert cholesterol to millimoles per liter, multiply by 0.0259; hemoglobin A_1c_ to proportion of total hemoglobin, multiply by 0.01.

Figure 2 shows the absolute HbA_1c_, LDL-C, and SBP levels as well as the percentages of participants with all 3 risk factors controlled across the 12-month intervention. The differences between the groups in mean HbA_1c_ and SBP levels were significant during the 12 months, whereas the group difference in LDL-C levels was not. Furthermore, there were no significant differences in the percentages of participants with all 3 risk factors controlled between the 2 groups at 6 and 12 months. In addition, changes in HbA_1c_, LDL-C, and SBP levels were similar for the 2 groups when assessed by subgroup (ie, sex, educational level, smoking or drinking status, BMI at baseline, and presence of hypercholesterolemia) (eTables 2-4 in Supplement 2). The reduction in HbA_1c_ levels was greater in participants younger than 65 years, whereas the reduction in SBP levels was greater in participants aged 65 years or older.

Daily total calorie intake in both groups decreased significantly from baseline to 6 and 12 months, particularly in the intervention group, but there was no statistically significant difference between the 2 groups (eTable 5 in Supplement 2). The proportions of carbohydrate, protein, and fat intake were similar in both groups. In addition, there were no significant differences in daily physical activity, MMAS scores, and physical and mental composite scores measured with the 12-item Short-Form Health Survey Questionnaire. The percentage of participants receiving glucose-lowering therapy and the number of antihyperglycemic used in the intervention group were significantly higher than those in the control group (eTable 6 in Supplement 2). By contrast, no significant differences were observed in the percentage of participants receiving BP-lowering or lipid-lowering therapy or the number of lipid-lowering agents.

### Adverse Events

Two deaths and 1 fracture event were reported during the trial. One participant in the control group started dialysis treatment during the 12-month trial. No other adverse events occurred in either group.

## Discussion

In this 12-month multicenter randomized clinical trial, a mobile message–based intervention significantly but modestly reduced HbA_1c_ and SBP levels compared with usual care. Mobile health interventions have gained much attention owing to affordability and ability to deliver health services, particularly in developing or low-income countries. Mobile phone text messages have potential to promote CVD risk factor control, but high-quality evidence regarding control of multiple CVD risk factors as primary outcomes is still limited. The TEXT-ME (Tobacco, Exercise and Diet Messages) study conducted in a single-center in Australia, showed that the use of a lifestyle-focused text messaging intervention compared with usual care resulted in a modest reduction in LDL-C level as a primary outcome, and moderate improvements in SBP, BMI and smoking status in patients with coronary heart disease (CHD) across 6 months.^15^ In the present trial, we developed a culturally adapted, content-enhanced mobile message–based module for a multifactor intervention in patients with diabetes and assessed the long-term common effect of the mobile message–based intervention on the control of multiple CVD risk factors. The text messaging intervention resulted in a statistically significant overall improvement in HbA_1c_, LDL-C, and SBP levels at 12 months in patients with uncontrolled T2D. A plausible mechanism underlying these interventions may be through more patient receipt intensive medication. This finding is encouraging for future studies of mobile text messaging to support secondary prevention goals in the management of diabetes. This finding may also have important public health and clinical importance for management of multiple risk factors in patients with diabetes.

Our findings are consistent with the existing evidence that supports the effectiveness of mobile phone text messaging for improving key CVD risk factors. A number of randomized clinical trials have shown improvements in weight loss, glycemic control, lipid, and BP lowering in diabetes and diagnosed CHD.^15,17,19,27^ In contrast, other studies have failed to detect significant effectiveness of mobile phone text messaging interventions in the improvement of SBP and LDL-C levels among patients with both diagnosed CHD and diabetes in China.^18,28^ One important difference between these 2 latter studies and the current study is that our study targeted multiple risk factors rather than individual risk factors, as the primary outcome was overall improvement in 3 risk factors (HbA_1c_, LDL-C, and SBP levels). In addition, our follow-up time was longer than that of all the aforementioned studies. For BP as individual outcome, our trial showed that the SBP level was significantly reduced in the mobile message–based intervention compared with usual care across 12 months. To our knowledge, this is the first trial to investigate the long-term effect of a mobile message–based intervention on BP control in patients with diabetes.

Numerous studies have assessed the mobile message–based intervention on glycemic control in patients with diabetes, but the results have been inconsistent.^14,16,17,18^ For instance, the text message–based diabetes self-management support program SMS4BG study reported that the intervention resulted in modest improvements in glycemic control in adults with uncontrolled diabetes.^29^ In contrast, the REACH (Rapid Education/Encouragement and Communications for Health) study showed a short-term effect of improved HbA_1c_ but no sustained effect at 15 months.^19^ One meta-analysis of 5 clinical trials showed no statistically significant effect of mobile text messaging on physical activity, glycemic control, weight, or BMI among patients with T2D.^30^ The discrepancy across studies may be due to small sample sizes, short intervention times (ie, 3-9 months), heterogeneity in the frequency of text message transmission, and different patterns of text message–based intervention. Our findings suggest that mobile text messaging helps to address the gap in evidence by showing that delivery of motivational reminders and health information via text message is possible and effective in diabetes management. Sahin et al^31^ conducted a systematic review and meta-analysis to explore optimal tailoring strategies, message design, and delivery features of mobile text messaging and reported that a direct and personalized communication and tailored interventions with 1-way messaging could be more effective than 2-way messages. In the present trial, we found that there was a modest reduction of 2.8% in HbA_1c_ in the intervention group, and a significant net difference of −0.3% between the groups at 12 months. The magnitude of improvement in HbA_1c_ observed in this study is consistent with the average reduction reported in previous studies,^14,16,18,32,33^ which would potentially limit the clinical value of nontailored and standardized text message intervention. Future large-scale, longer-term studies of tailored 1-way text message intervention are needed to determine its effectiveness in controlling cardiovascular risk factors in populations with T2D.

We did not detect differences between groups in LDL-C and BMI, which is consistent with the CHAT-DM (Cardiovascular Health and Text Messaging–Diabetes Mellitus) trial.^25^ Our study enrolled patients with diabetes although it did not restrict inclusion to individuals with uncontrolled hyperlipidemia, which may explain the lack of effect on LDL-C levels. Moreover, our data showed that there was no significant reduction in caloric intake or the proportion of fat intake in either group and no significant difference in lipid-lowering medication and physical activity between the 2 groups, which may explain the negative results. Future mobile message–based interventions should strengthen education in the adjustment of the diet structure, especially the control of fat intake and blood lipid management. Future studies are needed to further determine if the effects of text messaging intervention on the control of these risk factors can be enhanced and sustained, especially when integrated into clinical practice.

### Limitations

This study has limitations. First, the primary outcomes were changes in the levels of risk factors (HbA_1c_, LDL-C, and BP) rather than clinical outcomes. However, we can infer from the literature^34,35^ that changes in risk factors can affect future clinical outcomes if they are sustained. Second, the intervention only targeted patient-initiated behavior change; however, the geographic and clinical characteristics of the patients may have improved the effectiveness of the intervention. Interventions aimed at both patients and health care professionals could be helpful to minimize the gap between guidelines and clinical practice. Third, behavioral outcomes in this trial were assessed through either self-reported measures or structured investigator-administered questionnaires, which may be subject to recall bias. Fourth, the lack of systematic data on participants’ health care utilization frequency precluded analysis of potential text message intervention effects on clinical visit patterns. Additionally, this study did not determine the value of the intervention using a cost-effectiveness analysis. A future cost-effectiveness analysis is needed to determine the value of the intervention.

## Conclusions

In this randomized clinical trial of adults with uncontrolled T2D in China, a mobile message–based intervention resulted in a modest improvement in HbA_1c_ levels and BP among patients with uncontrolled T2D compared with usual care. These findings suggest that mobile message–based strategies for improving glycemic control and CVD risk factors should be considered for adults with T2D.

## Notes

Supplement 1.
Trial Protocol

Supplement 2. **eTable 1.** Primary and Secondary End Point Analyses at 6 and 12 Months of Follow-Up Using Multiple Imputation for Missing Data **eTable 2.** Changes in the HbA1c Levels by Subgroups at 12 Months of Follow-Up **eTable 3.** Changes in the LDL-C levels by Subgroups at 12 Months of Follow-Up **eTable 4.** Changes in the SBP Levels by Subgroups at 12 Months of Follow-Up **eTable 5.** Changes in the Medication Adherence, Dietary Intake and Physical Activity During the 12-Month Follow-Up **eTable 6.** Changes in the Proportions of Medication Recipient at 6 and 12 Months of Follow-Up **eFigure 1.** Absolute Waist Circumference, Weight, BMI and Diastolic BP Levels According to Randomization Group **eFigure 2.** Absolute Fasting Glucose, TC, TG and HDL-C Levels According to Randomization Group

Supplement 3.
Data Sharing Statement
