# Supplementary material for: Mobile App–Based Intervention and Cardiovascular Risk Factors in Patients With Uncontrolled Type 2 Diabetes: A Randomized Clinical Trial
Source: JAMA Netw Open. 2025 Sep 2;8(9):e2529762. doi: 10.1001/jamanetworkopen.2025.29762 (PMC12406060; doi:10.1001/jamanetworkopen.2025.29762)
Supplement: Supplement 1. — Trial Protocol [file jamanetwopen-e2529762-s001.pdf]

Study Protocol: Text messaging and Cardiovascular Health in Type 2 Diabetes

（TEACH Study）

Primary Investigator：Huijie Zhang M.D & Ph.D.,

Department of Endocrinology and Metabolism,

Nanfang Hospital, Southern Medical University

1. Background and aim

Diabetes has become a major public health issue around the world. Globally, the prevalence of diabetes is about 8.8% and the number of people with diabetes is about 425 million, which will rapidly rise to 629 million by 2045(1). The prevalence of diabetes is increasing dramatically in most countries around the world(1). According to a Chinese survey, the prevalence of diabetes in adults aged over 20 years increased from 2.5% in 1994 to 11.6% in 2013, which means about 114 million adults are suffering from diabetes (2). However, a study showed that the awareness, treatment, and control of diabetes were only 30.1%, 25.8% and 39.7%, respectively(2).

In the past 20 years, the total number of deaths attributed to diabetes has doubled(3), and the estimated diabetes-related death among people aged 20-79 is about 4.0 million in 2017(1). Diabetes is not only known as a major risk factor for cardiovascular diseases (CVD) but also a major cause of mortality (4-6). It is estimated that diabetes account for 21% and 13% respectively of deaths associated with CVD and stroke worldwide, and 84% of deaths induced by diabetes and CVD happen in low-income countries (7). This phenomenon is related to the poor control of diabetes and other risk factors of CVD. Most people living with diabetes are combined with multiple cardiovascular risk factors, such as obesity, hypertension and dyslipidemia, or with a history of atherosclerotic cardiovascular disease (ASCVD) (8; 9). A survey has found that 86% of Chinese adults with diabetes have at least one uncontrolled cardiovascular risk factor, such as hemoglobin A_{1c} (HbA_{1c}) ≥ 7.0%, blood pressure (BP) ≥ 140/90mm Hg, or low-density lipoprotein cholesterol (LDL-C) ≥ 100mg/dL, while 12.7%

of diabetes are complicated with ASCVD (10).

At present, many treatments, including lifestyle intervention, anti-platelet aggregation, lipid reduction and blood pressure control, have been proved to reduce cardiovascular events in patients with diabetes and are recommended in clinical guidelines (11). However, there are gaps between evidence and practice, especially lifestyle intervention, one of the most effective treatments (12), is often difficult to adhere to in clinical practice. The main reasons leading to this dilemma are lack of awareness and poor adherence (13-15). The education of the primary health service centers and increasing patient visits may help, but it is bound to greatly increase the current medical burden and costs. Therefore, seeking an efficient and universal strategy to improve lifestyle change and compliance is essential and urgent. Text messaging can remind, encourage and stimulate patients to manage the cardiovascular risk factors for diabetes, which theoretically improve their adherence and the treatment of diabetes (16-20). However, most existing studies have relatively small sample size, short intervention time (i.e., for 3–9 months), different patterns of text messaging intervention. Importantly, these existing clinical trials of mobile messaging-based interventions conducted in population with diabetes tended to focused only on glycemic control rather than multiple CVD risk factors control. Indeed, most patients with diabetes require long-term multifactorial therapy to simultaneous reduction of multiple risk factors because of its multiple comorbid conditions (21; 22). Therefore, it needs to evaluate mobile messaging-based interventions on reduction of CVD risk factors among patients with diabetes and fill the knowledge gaps.

In this randomized controlled trial, we aimed to evaluate the effectiveness of a mobile messaging-based intervention on cardiovascular risk factors management in patients with uncontrolled type 2 diabetes.

2. Research Design and Methods

2.1 Overall design

This study is a parallel, randomized, multicenter clinical trial with 800 participants in 5 hospitals in China with 12-months follow-up. Eligible participants with uncontrolled type 2 diabetes will be randomly assigned to the mobile messaging-based intervention or usual care for 12 months. The primary outcome comprised the mean changes in HbA1c levels, LDL-C levels, and systolic blood pressure (SBP) levels from baseline to 12

months simultaneously modeled for a single overall treatment effect.

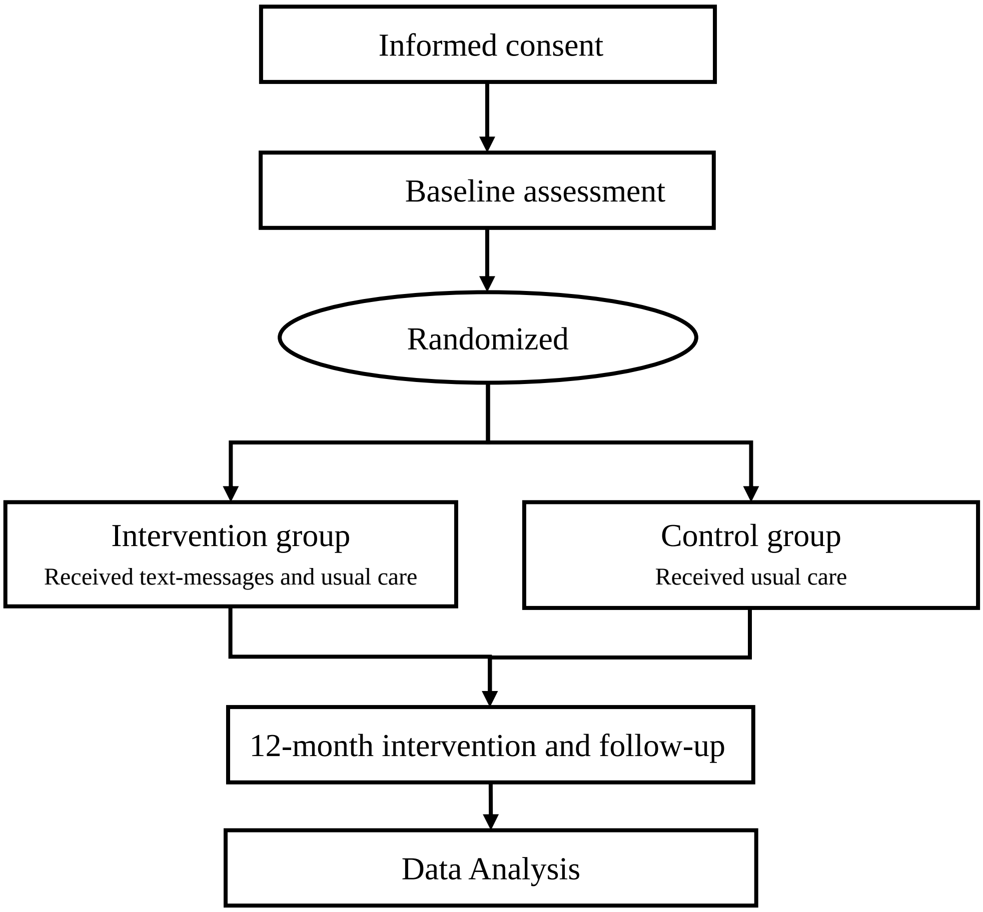

Figure 1. study design

2.2 Randomization and masking

Eligible participants were randomly assigned to either the mobile messaging-based intervention or control group in a 1:1 ratio. Randomization was stratified by study centers and performed centrally at the Research and Data Coordinating Center in Nanfang Hospital. The randomization schedules were generated using the PROC PLAN procedure in SAS statistical Software with a block size of six (Version 9.4). The research nurses, investigators, and statisticians were blinded to the treatment allocation. The clinical outcomes were also collected by blinded research doctors during the study period.

2.3 Study Population.

2.3.1 Inclusion criteria

Patients will be eligible if they are adults over 18 years old with poorly control Type 2 diabetes mellitus (defined as HbA1c ≥ 7% or if combined with clinical CVD ≥ 7.5%) comorbid with at least one risk factor for CVD (such as SBP ≥ 140 mmHg and / or diastolic blood pressure (DBP) ≥ 90 mmHg ≥ and / or LDL-C ≥

100mg/dL) or clinically diagnosed ASCVD (such as acute coronary syndrome, ischemic stroke, Transient ischemic attack or peripheral artery disease). All participants had access to a smartphone to read and accept text messages. All participants would provide written informed consent at the initial trial visit.

2.3.2 Exclusion criteria

Exclusion criteria included serious heart failure (NYHA [New York Heart Association] class III to IV), end-stage kidney disease, current or planned pregnancy, and inability to adhere to 12-month follow-up.

2.3.3 Termination criteria

Termination criteria: (1) Subjects with serious complications or severe adverse events; (2) Subjects are pregnant or treated with dialysis; (3) Subjects withdrawal the informed consent form, unwillingness to compliant to the intervention or other unexpected reasons.

2.4 Intervention.

All participants included in this study will receive usual care. Participants assigned to the mobile messaging-based intervention group will receive mobile text messaging that was regularly delivered by SMS (short messaging service) platform to remind, encourage and motivate patients to participate in the behaviors needed for improving glycaemic control and CVD risk factors management. About 500 text messages are included in this study, covering 7 aspects: general information about CVD and diabetes, healthy diet, physical activity, medical adherence, glucose monitoring, blood pressure control, smoking cessation. The content of text message is based on the recommendation from the Chinese Diabetes Society(23) and American Diabetes Association(24), a text mess bank in plain language was established and modified according to Chinese diet culture and language habits. 6 messages per week will be sent through the messaging platform during the 12-month study period. Researchers will save and checke the delivery log to ensure successful delivery. At each visit, participants in the intervention group are asked to confirm or update the phone number and complete a dedicated satisfaction survey questionnaire to investigate the reading proportion and satisfaction with the text messages

Table1. Text message examples

Category	Content/Explanation	Example
**General education about CVD and diabetes**	Basic information about CVD and diabetes	At present diabetes cannot be cured, but it can be controlled through a variety of treatment methods. It mainly includes five aspects: Diabetes self-management education and support, self-monitoring of blood glucose, healthy diet, physical activity and drug therapy.
**Healthy diet**	Encourage and guide patients to form a healthy diet and eating habits	Foods high in fiber or with a lower glycemic index or glycemic load, such as vegetables, fruits, soy products, whole grains, and dairy products without added sugar, is more recommended for people with diabetes.
**Physical activity**	Encourage and guide patients to exercise	Start exercising as a part of your daily life. Spend 20-30 minutes every day to do some exercise. We suggest you start with a brisk walk, which is simple but helpful!
**Medication adherence**	Remind medication and popularize right knowledge about medication	Regularly taking oral hypoglycemic drugs or injecting insulin is necessary for glucose control. Did you forget to take your medicine? Never mind, set a repetitive alarm on your phone to remind you.
**Glucose monitoring**	Remind glucose monitoring and prevent hypoglycemia	Glucose monitoring is an important way to check your body, helping you assess whether you need to adjust your medication and lifestyle recently.
**Blood pressure control**	Remind blood pressure control and management	The main goal of antihypertensive treatment is to control blood pressure. The ultimate goal of antihypertensive therapy is to minimize the incidence and mortality of cardiocerebrovascular diseases in patients with hypertension.
**Smoking Cessation**	Encourage and help smoking cessation	Smoking is harmful! Nicotine can stimulate adrenaline secretion, directly causing high blood pressure and glucose fluctuations.

2.5 Outcomes

2.5.1 Primary outcome

The primary outcome comprised the mean changes in HbA1c levels, LDL-C levels, and SBP levels from baseline to 12 months simultaneously modeled for a single overall treatment effect.

2.5.2 Secondary outcomes

- Change in HbA1c levels
- Change in fasting blood glucose (FBG) levels
- Change in lipid levels, included total cholesterol (TC) levels, triglycerides (TG) levels, high-density

lipoprotein cholesterol (HDL-C) levels

- Change in blood pressure levels
- Change in body-mass index (BMI).
- Proportion of participants with controlled HbA1c, LDL-C, and BP levels at 12 months.
- Change in self-reported medication adherence measured by the Morisky medication adherence scale (MMAS).
- Change in health-related quality of life measured according to the 12-item Short-Form Health Survey Questionnaire.

2.6 Baseline and follow-up visits

2.6.1 Screening/baseline visit

The purpose of the screening / baseline visit is to confirm patients' eligibility and interest in the proposed study, and obtain informed consent, sociodemographic factors (age, sex, education, employment status), lifestyle habits (smoking status, drinking status, physical activity, diet), medications and medical history, physical measurements, and laboratory measurements. Body weight, height, waist circumference and blood pressure will be measured by trained staff using standard procedures. Fasting blood samples will be collected at baseline visit. Laboratory measurements include HbA1c, fasting blood glucose and lipids.

2.6.2. Follow-up and termination visits

Physical measurements data and laboratory measurements will be collected by the study staff every 3 month during the 12-month intervention. Information on lifestyle habits (smoking status, drinking status, physical activity, diet), medical record data and medications will be scheduled at 6, 12 months following the baseline visit. In addition, adverse events if happen will be confirmed by the researchers based on self-reported questionnaires and medical records.

Table 2．Study visits and data collection

	S/R	M1	M2	M3	M6	M9	M12
Inform consent	X						

Randomization	X						
Demographic information	X						
Medica history and medications	X				X		X
Lifestyle questionnaire	X				X		X
Blood pressure	X	X	X	X	X	X	X
Body weight, waist circumference	X	X	X	X	X	X	X
HbA1c	X			X	X	X	X
Blood lipids	X			X	X	X	X
Blood glucose	X			X	X	X	X
Blood samples	X				X		X
Adverse event	X	X	X	X	X	X	X

S/R: Screening and randomization

2.7 Measurements

2.7.1 Questionnaire

Information on Information on age, sex, education level, smoking status, physical activity, diet, smoking status, drinking status, medical history (such as diabetes, hypertension, dyslipidemia, CVD), medication use, medication adherence and health-related quality of life will be collected from the standardized questionnaire. Medication adherence will be measured by the Morisky medication adherence scale (MMAS) (range, 0-8, with lower scores indicating lower adherence). 12-item Short-Form Health Survey Questionnaire will be used to obtain the health-related quality of life (25).

2.7.2 Physical measurements

The blood pressure of each participant will be measured three times at each visit by blinded research doctors or nurses using an automatic electronic sphygmomanometer (OMRON Company). During each visit, participants will be asked to rest for 5 minutes and take the measurement in a sitting position, and they should avoid alcohol, cigarettes, coffee or tea, and physical exercise for 30 minutes before blood pressure measurement.

Height, weight, and waist circumference will also be measured by the trained staff according to standard procedures.

2.7.3 Laboratory measurements

Blood samples will be collected to measure the level of blood glucose, HbA1c and blood lipids after 10 hours of fasting. The plasma glucose concentration will be determined by hexokinase mediated reaction. HbA1c levels will be measured by High Performance Liquid Chromatography (HPLC). Automatic biochemical analyzer will be used to measure serum total cholesterol, LDL-C, HDL-C, and triglycerides. All measurements will be conducted at Guangzhou Kinmed Center for Clinical Laboratory.

3. Adverse events

3.1 Definition of adverse events

Adverse events (AE) are defined as the occurrence of new diagnosis or deterioration of diseases during the treatment. The causal relationship to the intervention will be further evaluated.

3.2 Definition of serious adverse events

Severe adverse events (SAE) are defined as the occurrence of any acute life-threatening event, a hospitalization for any cause other than routine delivery, prolonged or permanent disability.

3.3 Surveillance and recording

Protection of participants from risks related to the intervention is of paramount concern to the physicians. All participants will be evaluated for their medical history before enrollment. Contact information of the physicians is available for all the participants.

All participants should be contacted regularly to evaluate the occurrence, severity, and duration of physical discomfort during intervention. Evidence of the occurrence of adverse events should be based on participants' self-report rather than suggestive questioning. Any medical adverse events will be recorded and the nature, severity, and cause-effect relationship will be evaluated by physicians and recorded in a timely manner on case report forms.

3.4 Assessment of severity

The severity of adverse events is assessed as follows:

Mild: usually temporary, and not affecting daily activities;

Moderate: causing discomfort and affecting normal activities, thought tolerable and not requiring participants to take any medication.

Severe: disrupting daily activities and intolerable, requiring participants to take medication immediately.

With regard to the causal-effect relationship, medical adverse events are divided into five categories: definite, probable, possible, unlikely, and unrelated. Only “definite” and “probable” are counted as adverse events.

3.5 Treatment

Any adverse events happen, appropriate care or medical treatment (if necessary) should be immediately provided to keep the safety of all participants. In addition, all adverse events and serious adverse events and treatment should be recorded and reported to the Principal Investigator and the Committee within 24 hours and complete the case report form.

4. Data management

4.1. Case report form

All case report forms for each participant should be filled out by study staff in a timely manner. The case report form should be double-checked for potential errors or missing data prior to patients leaving the clinic. All data, including screening assessments, questionnaires, physical examinations, and laboratory examinations, will be filed in the participant’s chart. Original documents, participants’ charts, and CRF forms will be stored in the study office.

4.2. Data entry

All data will be double-entered by researcher staff. Two sets of databases will be generated and tested for consistency using the SAS program. Whenever inconsistencies are found, the data will be corrected by re-examination of the original case report forms or laboratory reports.

4.3 Data reports

Several standardized reports will be generated as follows: 1) participant recruitment and follow-up; 2)

demographics; 3) data quality and monitoring; 4) adverse events. These reports will be used for study management. These reports will be blinded to research personnel who collect study outcomes.

5. Statistics

5.1 Estimation of sample size

The study will enroll 800 participants, who will be randomly allocated to the mobile messaging-based intervention or control group in a 1:1 ratio. The primary outcome comprised the mean changes in HbA1c levels, LDL-C levels, and SBP levels from baseline to 12 months simultaneously modeled for a single overall treatment effect(26). We anticipate 80% statistical power to detect a 5% difference of the net change in standardized overall treatment effect at a significance level of 0.05 using a 2-tailed test; this corresponds to detect a detectable difference of 0.5% in HbA1c change (7.2±1.6%), 5 mm Hg in systolic blood pressure (132±18 mmHg at baseline) and 0.25 mmol/L in LDL-C (2.94±0.96 mmol/L)(27-29). Assuming a dropout rate of 20% over 12 months, we will recruit 400 subjects to each group in the proposed clinical trial.

5.2.1 Data analysis plan

Data was analyzed according to participants' randomization assignments, regardless of their subsequent status (intention to-treat principle). All the analyses will be conducted using SAS version 9.4 (SAS Institute Inc). Group differences in the trial outcomes will be evaluated using the general linear model for continuous variables and the chi-square test for categorical variables. Continuous variables will be presented as mean ± standard deviation (SD) or median (interquartile range), and categorical variables will be presented as frequencies with percentages. In this study, the overall intervention effect on HbA1c, LDL-C, and SBP will be tested using a scaled marginal model, in which each outcome was scaled by its SD. Generalized estimating equations will be used to calculate the regression coefficient and outcome-specific scale variables in SAS PROC GENMOD, and the assumption of an overall intervention effect will be tested using a score test(30). Group differences for the secondary outcomes will be also evaluated with the use of the generalized estimating equations for continuous variables adjusting for baseline levels corresponding to each study outcome. In addition, the correlations of outcomes among participants within each clinic center using an exchangeable

correlation structure will be also taken into account in the analysis. Multiple imputations for missing data in the multivariable analyses will be conducted using the Markov chain Monte Carlo method.

Subgroup analyses of HbA1c, LDL-C, and SBP will be also conducted according to age (≤60 and >60 years), sex (male and female), education levels (junior high school degree or below, high school education or above), employment status (never worked/unemployed, retired and on duty), smoking status (never, former and current), current drinking (yes or no), prevalent obesity (yes or no), prevalent hypertension (yes or no), prevalent hypercholesterolemia (yes or no). A two-sided P value less than 0.05 is considered statistically significant.

6. Quality control

The proposed trial will comply with China's Standard for Quality Management of Clinical Trials (GCP) and other relevant laws and regulations on clinical trials. Participants' personal information will be confidential and will not be disclosed without permission.

The standard quality control process will be carried out at each step of the study, including repeated laboratory measurements in 5-10% of random blind samples, and all data will be entered independently.

Establish a research procedure manual (MOP): we will develop a standardized process of data collection, which will include the recruitment of subjects, instructions for the use of tables, intervention programs, index measurement, specimen collection and management, etc., to guide all tabular information entry and diagnosis and treatment processes, and other aspects of the study.

Training: all researchers will receive relevant training before the start of the study, including standardized processes, recruitment processes, follow-up, intervention programs, measurement procedures, and the use of MOP. At the same time, regular retraining sessions will be conducted to ensure the quality of research.

Quality monitoring and reporting: quality control personnel will regularly review the QC data, including the timeliness of the completion of the visit, data collection, program implementation and data quality.

A steering committee (SC), chaired by Prof. Huijie Zhang, Nanfang Hospital, Southern Medical University, is responsible for the study. The SC is responsible for organizing and supervising the research implementation, approving the research program, including significant revisions, monitoring subject recruitment and retention,

delivering of intervention programs, data collection, quality control, and respond to questions raised by the institutional review board (IRB). The research center will play a critical role in the patient recruitment, randomization, delivery of intervention, data collection, quality control, data analysis, and the report and publication of the research results.

In order to facilitate communication among investigators and research staff, regular working meetings will be held. The SC will discuss and make decisions on relevant scientific and management issues in the study with three live meetings a year and monthly conference calls. Important information, such as recruitment yields, data completion and quality, and adherence to the protocol, will be sent to committee members and core staff by e-mail monthly.

7. Ethics Approval

The proposed trial complies with the Helsinki Declaration (1996 edition) and the relevant regulations of Chinese clinical trials. The study protocol and informed consent form are approved by institutional review boards of the Nanfang Hospital of Southern Medical University before implementation.

All revisions on protocol or informed consent (excluding administrative changes and revisions that have no impact on the participants, implementation of trial, or data) must be submitted to the Ethics Committee for approval prior to the implementation of amendments. Serious adverse events must be reported to the Ethics Committee as required by the ethics committee.

Before enrollment, the informed consent is accessible to the patients. The background, aim, methods and measurements of the trial are introduced thoroughly by the researcher. Individual rights and obligations, possible risks and benefits during the intervention must be explained in detail. Researchers are responsible to answer questions raised by patients accurately in plain language. Patients are allowed to discuss with family members and decide whether to participate in the study. All subjects approve the collection and use of the trial data and are willing to participate the follow-up. Researchers should obtain the permission of the participants to use the collected data if they withdraw. Participants will be required to sign informed consent before enrollment. Informed consent will be kept as original materials for future reference. The study will be registered on the NIH

Clinical Trial website before enrollment.

8. Proposed study timeline

The implementation of the trial is expected to take about 2.5 years after registration in the clinicaltrials.gov the trial, in which MOP development, personnel training, and subject recruitment take about 1 year. Intervention and data collection take about 1 year, data entry, data analysis and manuscript preparing take about 0.5 years.

References

1. Federation ID. IDF Diabetes Atlas 2017. 2017;

2. Y X, L W, J H, et al. Prevalence and control of diabetes in Chinese adults. JAMA 2013;310:948-959

3. R L, M N, K F, et al. Global and regional mortality from 235 causes of death for 20 age groups in 1990 and 2010: a systematic analysis for the Global Burden of Disease Study 2010. Lancet (London, England) 2012;380:2095-2128

4. CS F, S C, PD S, et al. Increasing cardiovascular disease burden due to diabetes mellitus: the Framingham Heart Study. Circulation 2007;115:1544-1550

5. AS G, D M, VL R, et al. Heart disease and stroke statistics--2014 update: a report from the American Heart Association. Circulation 2014;129:e28-e292

6. FB H, MJ S, CG S, et al. The impact of diabetes mellitus on mortality from all causes and coronary heart disease in women: 20 years of follow-up. Archives of internal medicine 2001;161:1717-1723

7. G D, CM L, S VH, et al. Global and regional mortality from ischaemic heart disease and stroke attributable to higher-than-optimum blood glucose concentration: comparative risk assessment. Lancet (London, England) 2006;368:1651-1659

8. SM G, JI C, SR D, et al. Diagnosis and management of the metabolic syndrome: an American Heart Association/National Heart, Lung, and Blood Institute Scientific Statement. Circulation 2005;112:2735-2752

9. D G, A G, P M, et al. Prevalence of cardiovascular disease risk factor clustering among the adult population of China: results from the International Collaborative Study of Cardiovascular Disease in Asia (InterAsia). Circulation 2005;112:658-665

10. ZJ Y, J L, JP G, et al. Prevalence of cardiovascular disease risk factor in the Chinese population: the 2007-2008 China National Diabetes and Metabolic Disorders Study. European heart journal 2012;33:213-220

11. JJ C, EL J, S L, et al. Cardiovascular Disease and Risk Management: Review of the American Diabetes Association Standards of Medical Care in Diabetes 2018. Annals of internal medicine 2018;168:640-650

12. Long-term effects of lifestyle intervention or metformin on diabetes development and microvascular

complications over 15-year follow-up: the Diabetes Prevention Program Outcomes Study. The lancet Diabetes & endocrinology 2015;3:866-875

13. JC C, M H, U S, et al. Physicians' perceptions of barriers to cardiovascular disease risk factor control among patients with diabetes: results from the translating research into action for diabetes (TRIAD) study. Journal of the American Board of Family Medicine : JABFM 2010;23:171-178

14. S N, C C, NA S, et al. Barriers to diabetes management: patient and provider factors. Diabetes research and clinical practice 2011;93:1-9

15. M P, RR R, T L, et al. Patient and provider perceptions of care for diabetes: results of the cross-national DAWN Study. Diabetologia 2006;49:279-288

16. CK C, J R, GS H, et al. Effect of Lifestyle-Focused Text Messaging on Risk Factor Modification in Patients With Coronary Heart Disease: A Randomized Clinical Trial. JAMA 2015;314:1255-1263

17. AL F, LC G, MI G, et al. Dulce Digital: An mHealth SMS-Based Intervention Improves Glycemic Control in Hispanics With Type 2 Diabetes. Diabetes care 2017;40:1349-1355

18. VL F, A G, A W, et al. Patients' engagement with "Sweet Talk" - a text messaging support system for young people with diabetes. Journal of medical Internet research 2008;10:e20

19. KT M, A R, V I, et al. Comprehensive approach for hypertension control in low-income populations: rationale and study design for the hypertension control program in Argentina. The American journal of the medical sciences 2014;348:139-145

20. LG P, J H-E, ML C, et al. A text messaging intervention to promote medication adherence for patients with coronary heart disease: a randomized controlled trial. Patient education and counseling 2014;94:261-268

21. Gaede P, Lund-Andersen H, Parving H-H, et al. Effect of a multifactorial intervention on mortality in type 2 diabetes. N Engl J Med 2008;358:580-591

22. Janssen PG, Gorter KJ, Stolk RP, et al. Randomised controlled trial of intensive multifactorial treatment for cardiovascular risk in patients with screen-detected type 2 diabetes: 1-year data from the ADDITION Netherlands study. Br J Gen Pract 2009;59:43-48

23. Weng J, Ji L, Jia W, et al. Standards of care for type 2 diabetes in China. Diabetes/Metabolism Research and Reviews 2016;32:442-458

24. Association AD. 4. Lifestyle Management: Standards of Medical Care in Diabetes-2018. Diabetes Care 2018;41:S38-S50

25. J W, M K, SD K. A 12-Item Short-Form Health Survey: construction of scales and preliminary tests of reliability and validity. Medical care 1996;34:220-233

26. J R, X L, LM R. Scaled marginal models for multiple continuous outcomes. Biostatistics (Oxford, England) 2003;4:371-383

27. Chow CK, Redfern J, Hillis GS, et al. Effect of Lifestyle-Focused Text Messaging on Risk Factor Modification in Patients With Coronary Heart Disease: A Randomized Clinical Trial. JAMA 2015;314:1255-1263

28. Dobson R, Whittaker R, Jiang Y, et al. Effectiveness of text message based, diabetes self management support programme (SMS4BG): two arm, parallel randomised controlled trial. BMJ 2018;361:k1959

29. Huo X, Spatz ES, Ding Q, et al. Design and rationale of the Cardiovascular Health and Text Messaging (CHAT) Study and the CHAT-Diabetes Mellitus (CHAT-DM) Study: two randomised controlled trials of text messaging to improve secondary prevention for coronary heart disease and diabetes. BMJ Open 2017;7:e018302

30. Roy J, Lin X, Ryan LM. Scaled marginal models for multiple continuous outcomes. Biostatistics 2003;4:371-383
