# Supplementary material for: Mobile App–Based Intervention and Cardiovascular Risk Factors in Patients With Uncontrolled Type 2 Diabetes: A Randomized Clinical Trial
Source: JAMA Netw Open. 2025 Sep 2;8(9):e2529762. doi: 10.1001/jamanetworkopen.2025.29762 (PMC12406060; doi:10.1001/jamanetworkopen.2025.29762)
Supplement: Supplement 2. — eTable 1. Primary and Secondary End Point Analyses at 6 and 12 Months of Follow-Up Using Multiple Imputation for Missing Data eTable 2. Changes in the HbA1c Levels by Subgroups at 12 Months of Follow-Up eTable 3. Changes in the LDL-C levels by Subgroups at 12 Months of Follow-Up eTable 4. Changes in the SBP Levels by Subgroups at 12 Months of Follow-Up eTable 5. Changes in the Medication Adherence, Dietary Intake and Physical Activity During the 12-Month Follow-Up eTable 6. Changes in the Proportions of Medication Recipient at 6 and 12 Months of Follow-Up eFigure 1. Absolute Waist Circumference, Weight, BMI and Diastolic BP Levels According to Randomization Group eFigure 2. Absolute Fasting Glucose, TC, TG and HDL-C Levels According to Randomization Group [file jamanetwopen-e2529762-s002.pdf]

# Supplementary Online Content

Zhang PZ, Guo D, Liu CQ, et al. Mobile app-based intervention and cardiovascular risk factors in patients with uncontrolled type 2 diabetes: a randomized clinical trial. *JAMA Netw Open*. 2025;8(9):e2529762. doi:10.1001/jamanetworkopen.2025.29762

**eTable 1.** Primary and Secondary End Point Analyses at 6 and 12 Months of Follow-Up Using Multiple Imputation for Missing Data
**eTable 2.** Changes in the HbA1c Levels by Subgroups at 12 Months of Follow-Up
**eTable 3.** Changes in the LDL-C levels by Subgroups at 12 Months of Follow-Up
**eTable 4.** Changes in the SBP Levels by Subgroups at 12 Months of Follow-Up
**eTable 5.** Changes in the Medication Adherence, Dietary Intake and Physical Activity During the 12-Month Follow-Up
**eTable 6**. Changes in the Proportions of Medication Recipient at 6 and 12 Months of Follow-Up
**eFigure 1.** Absolute Waist Circumference, Weight, BMI and Diastolic BP Levels According to Randomization Group
**eFigure 2.** Absolute Fasting Glucose, TC, TG and HDL-C Levels According to Randomization Group

This supplementary material has been provided by the authors to give readers additional information about their work.

**eTable 1. Primary and Secondary End Point Analyses at 6 and 12 Months of Follow-Up Using Multiple Imputation for Missing Data**

| Study outcomes | Intervention group (N=410) | Control group (N=409) | Difference between Groups[a] | *P* value | Co-*P* value |
|---|---|---|---|---|---|
| **Primary outcomes at 12 months*** | | | | | |
| Change in hemoglobin A1c, % | -2.8 (-2.9 to -2.6) | -2.5 (-2.7 to -2.3) | -0.2 (-0.5 to -0.0) | **.03** | |
| Change in LDL-C, mg/dL | -11.4 (-15.1 to -7.7) | -12.1 (-16.1 to -8.1) | 0.7 (-4.7 to 6.2) | .80 | **<.001** |
| Change in systolic blood pressure, mmHg | -2.4 (-3.7 to -1.0) | -0.0 (-1.4 to 1.4) | -2.3 (-4.3 to -0.4) | **.02** | |
| **Primary outcomes at 6 months*** | | | | | |
| Change in hemoglobin A1c, % | -3.1 (-3.3 to -2.9) | -2.7 (-2.9 to -2.5) | -0.4 (-0.6 to -0.2) | **<.001** | |
| Change in LDL-C, mg/dL | -11.0 (-14.9 to -7.2) | -14.4 (-18.3 to -10.5) | 3.4 (-2.0 to 8.7) | .22 | **<.001** |
| Change in systolic blood pressure, mmHg | -3.2 (-4.5 to -1.8) | -1.2 (-2.5 to 0.2) | -2.0 (-4.0 to -0.1) | **.04** | |
| **Secondary outcomes** | | | | | |
| Proportion of controlled hemoglobin A1c, % | 210 (51.2) | 166 (40.6) | / | **.002** | |
| Proportion of LDL-C <100 mg/dL, % | 130 (31.7) | 126 (30.8) | / | .78 | |
| Proportion of blood pressure<140/90 mmHg, % | 335 (81.7) | 325 (79.5) | / | .42 | |
| Change in fasting plasma glucose, mg/dL | | | | | |
| 6 months | -42.3 (-47.7 to -36.9) | -31.4 (-37.1 to -25.4) | -11.0 (-18.9 to -3.1) | **.007** | |
| 12 months | -32.8 (-38.0 to -27.4) | -27.4 (-32.8 to -22.0) | -5.4 (-13.0 to 2.2) | .16 | |
| Change in total cholesterol, mg/dL | | | | | |
| 6 months | -16.9 (-21.6 to -12.2) | -16.5 (-21.3 to -11.7) | -0.4 (-7.0 to 6.3) | .92 | |
| 12 months | -13.3 (-17.9 to -8.7) | -14.5 (-19.5 to -9.6) | 1.2 (-5.4 to 7.8) | .72 | |
| Change in triglycerides, mg/dL | | | | | |
| 6 months | -28.9 (-46.1 to -11.6) | -14.1 (-31.8 to 3.7) | -14.8 (-39.7 to 10.1) | .24 | |
| 12 months | -15.7 (-33.3 to 1.9) | -12.7 (-30.2 to 4.8) | -3.0 (-27.8 to 21.8) | .81 | |
| Change in HDL-C, mg/dL | | | | | |

© 2025 Zhang PZ et al. *JAMA Network Open*.

| | | | | |
|---|---|---|---|---|
| 6 months | 2.9 (2.0 to 3.9) | 3.2 (2.2 to 4.1) | -0.2 (-1.6 to 1.1) | .73 |
| 12 months | 3.1 (2.2 to 4.1) | 3.2 (2.2 to 4.2) | -0.1 (-1.4 to 1.3) | .92 |
| Change in diastolic blood pressure, mmHg | | | | |
| 6 months | -1.9 (-2.7 to -1.0) | -1.0 (-1.8 to -0.1) | -0.9 (-2.1 to 0.3) | .13 |
| 12 months | -1.4 (-2.3 to 0.6) | -0.7 (-1.6 to 0.2) | -0.7 (-1.9 to 0.5) | .23 |
| Change in body-mass index, kg/m$^2$ | | | | |
| 6 months | -0.2 (-0.4 to -0.1) | -0.2 (-0.4 to -0.0) | -0.0 (-0.3 to 0.2) | .77 |
| 12 months | -0.1 (-0.3 to 0.1) | -0.0 (-0.2 to 0.1) | -0.0 (-0.3 to 0.2) | .72 |

Abbreviation: HDL-C, high-density lipoprotein cholesterol; LDL-C, low-density lipoprotein cholesterol.

[a] The difference in single study outcome between groups was tested using a mixed-effects model in PROC MIXED, with randomized treatment as a factor and baseline end point value or clinic center as a covariate using a multiple imputation approach for missing data.

© 2025 Zhang PZ et al. *JAMA Network Open*.

**eTable 2. Changes in the HbA1c Levels by Subgroups at 12 Months of Follow-Up**

| Study outcomes | N, % | Intervention group (N=410) | Control group (N=409) | Difference between Groups | *P* for interaction |
|---|---|---|---|---|---|
| **Age, years** | | | | | |
| < 65 | 728 (88.9) | -2.9 (-3.1 to -2.7) | -2.6 (-2.7 to -2.4) | -0.3 (-0.6 to -0.1) | **.04** |
| ≥ 65 | 91 (11.1) | -1.6 (-2.0 to -1.1) | -2.0 (-2.4 to -1.5) | 0.4 (-0.3 to 1.0) | |
| **Sex** | | | | | |
| Male | 552 (67.4) | -3.1 (-3.3 to -2.9) | -2.8 (-3.0 to -2.6) | -0.3 (-0.6 to 0.0) | .99 |
| Female | 267 (32.6) | -2.1 (-2.4 to -1.8) | -1.9 (-2.2 to -1.6) | -0.2 (-0.6 to 0.2) | |
| **Education levels** | | | | | |
| Junior high school degree or below | 379 (46.3) | -2.6 (-2.8 to -2.3) | -2.4 (-2.6 to -2.1) | -0.2 (-0.5 to 0.1) | .70 |
| High school education or above | 440 (53.7) | -2.9 (-3.1 to -2.7) | -2.6 (-2.9 to -2.4) | -0.3 (-0.6 to 0.0) | |
| **Current smoking** | | | | | |
| Yes | 239 (29.2) | -3.1 (-3.4 to -2.8) | -2.5 (-2.9 to -2.2) | -0.6 (-1.0 to -0.1) | .11 |
| No | 580 (70.8) | -2.6 (-2.8 to -2.4) | -2.5 (-2.7 to -2.3) | -0.1 (-0.4 to 0.1) | |
| **Current drinking** | | | | | |
| Yes | 136 (16.6) | -3.0 (-3.4 to -2.6) | -2.7 (-3.1 to -2.3) | -0.3 (-0.9 to 0.2) | .86 |
| No | 683 (83.4) | -2.7 (-2.9 to -2.5) | -2.5 (-2.7 to -2.3) | -0.3 (-0.5 to 0.0) | |
| **Obesity** | | | | | |
| Yes | 132 (16.1) | -2.2 (-2.6 to -1.7) | -2.3 (-2.7 to -1.9) | 0.1 (-0.5 to 0.7) | .31 |
| No | 687 (83.9) | -2.9 (-3.1 to -2.7) | -2.5 (-2.7 to -2.3) | -0.3 (-0.6 to -0.1) | |
| **Current hypertension** | | | | | |
| Yes | 281 (34.3) | -2.4 (-2.7 to -2.1) | -2.3 (-2.6 to -2.0) | -0.1 (-0.5 to 0.3) | .38 |
| No | 538 (65.7) | -2.9 (-3.1 to -2.7) | -2.6 (-2.8 to -2.4) | -0.3 (-0.6 to -0.1) | |
| **Current hypercholesterolemia** | | | | | |
| Yes | 696 (85.0) | -2.9 (-3.0 to -2.7) | -2.6 (-2.8 to -2.4) | -0.2 (-0.5 to 0.0) | .82 |
| No | 123 (15.0) | -2.3 (-2.7 to -1.8) | -1.9 (-2.4 to -1.4) | -0.4 (-1.0 to 0.3) | |

© 2025 Zhang PZ et al. *JAMA Network Open*.

The difference in single study outcome between groups was tested using the mixed-effects model in PROC MIXED, with randomized treatment as a factor and baseline end-point value and clinic center as a covariate using a multiple imputation approach for missing data.

© 2025 Zhang PZ et al. *JAMA Network Open*.

**eTable 3. Changes in the LDL-C levels by Subgroups at 12 Months of Follow-Up**

| Study outcomes | N, % | Intervention group (N=410) | Control group (N=409) | Difference between Groups | *P* for interaction |
|---|---|---|---|---|---|
| **Age, years** | | | | | |
| < 65 | 728 (88.9) | -11.9 (-15.8 to -8.1) | -11.0 (-15.1 to -6.9) | -0.9 (-6.6 to 4.7) | .06 |
| ≥ 65 | 91 (11.1) | -5.2 (-17.0 to 6.6) | -17.8 (-29.8 to -5.9) | 12.6 (-4.2 to 29.5) | |
| **Sex** | | | | | |
| Male | 552 (67.4) | -9.6 (-14.0 to -5.3) | -8.2 (-13.0 to -3.4) | -1.4 (-7.9 to 5.1) | .48 |
| Female | 267 (32.6) | -14.3 (-21.1 to -7.5) | -19.0 (-25.8 to -12.3) | 4.8 (-4.8 to 14.3) | |
| **Education levels** | | | | | |
| Junior high school degree or below | 379 (46.3) | -14.4 (-20.2 to -8.5) | -17.5 (-23.0 to -11.9) | 3.1 (-4.9 to 11.1) | .82 |
| High school education or above | 440 (53.7) | -8.9 (-13.5 to -4.2) | -6.3 (-11.7 to -0.8) | -2.6 (-9.8 to 4.6) | |
| **Smoking status** | | | | | |
| Yes | 239 (29.2) | -6.9 (-13.7 to -0.1) | -2.4 (-10.2 to 5.3) | -4.4 (-14.7 to 5.8) | .19 |
| No | 580 (70.8) | -12.9 (-17.3 to -8.5) | -15.5 (-20.0 to -11.0) | 2.6 (-3.7 to 8.9) | |
| **Current drinking** | | | | | |
| Yes | 136 (16.6) | -9.5 (-18.4 to -0.7) | -8.2 (-18.0 to 1.6) | -1.3 (-14.5 to 11.9) | .99 |
| No | 683 (83.4) | -11.5 (-15.5 to -7.4) | -12.6 (-16.9 to -8.4) | 1.2 (-4.7 to 7.0) | |
| **Obesity** | | | | | |
| Yes | 132 (16.1) | -4.9 (-13.6 to 3.7) | -10.2 (-19.3 to -1.2) | 5.3 (-7.2 to 17.8) | .33 |
| No | 687 (83.9) | -12.3 (-16.3 to -8.2) | -12.4 (-16.7 to -8.0) | 0.1 (-5.8 to 6.0) | |
| **Current hypertension** | | | | | |
| Yes | 281 (34.3) | -9.4 (-16.2 to -2.7) | -13.0 (-20.0 to -5.8) | 3.5 (-6.3 to 13.3) | .27 |
| No | 538 (65.7) | -11.9 (-16.3 to -7.6) | -11.5 (-16.1 to -6.8) | -0.5 (-6.8 to 5.9) | |
| **Current hypercholesterolemia** | | | | | |
| Yes | 696 (85.0) | -16.4 (-20.5 to -12.2) | -16.4 (-20.8 to -12.1) | 0.0 (-6.0 to 6.0) | .98 |
| No | 123 (15.0) | 18.9 (11.4 to 26.4) | 13.4 (4.9 to 21.9) | 5.5 (-5.9 to 16.8) | |

© 2025 Zhang PZ et al. *JAMA Network Open*.

The difference in single study outcome between groups was tested using the mixed-effects model in PROC MIXED, with randomized treatment as a factor and baseline end-point value and clinic center as a covariate using a multiple imputation approach for missing data.

© 2025 Zhang PZ et al. *JAMA Network Open*.

**eTable 4. Changes in the SBP Levels by Subgroups at 12 Months of Follow-Up**

| Study outcomes | N, % | Intervention group (N=410) | Control group (N=409) | Difference between Groups | *P* for interaction |
|---|---|---|---|---|---|
| **Age, years** | | | | | |
| < 65 | 728 (88.9) | -2.5 (-3.8 to -1.1) | -0.5 (-2.0 to 0.9) | -1.9 (-3.9 to 0.0) | **.02** |
| ≥ 65 | 91 (11.1) | -2.6 (-8.0 to 2.7) | 2.0 (-3.5 to 7.4) | -4.6 (-12.2 to 3.0) | |
| **Sex** | | | | | |
| Male | 552 (67.4) | -2.5 (-4.1 to -1.0) | -0.8 (-2.5 to 0.8) | -1.7 (-4.0 to 0.5) | .17 |
| Female | 267 (32.6) | -2.2 (-5.0 to 0.5) | 1.2 (-1.6 to 4.0) | -3.5 (-7.4 to 0.5) | |
| **Education levels** | | | | | |
| Junior high school degree or below | 379 (46.3) | -1.0 (-3.2 to 1.2) | -0.7 (-2.8 to 1.4) | -0.3 (-3.3 to 2.8) | .19 |
| High school education or above | 440 (53.7) | -3.5 (-5.2 to -1.8) | 0.4 (-1.6 to 2.4) | -3.9 (-6.5 to -1.3) | |
| **Current smoking** | | | | | |
| Yes | 239 (29.2) | -1.8 (-4.3 to 0.7) | -2.5 (-5.4 to 0.4) | 0.7 (-3.1 to 4.5) | .09 |
| No | 580 (70.8) | -2.8 (-4.4 to 1.2) | 0.7 (-1.0 to 2.3) | -3.5 (-5.8 to -1.2) | |
| **Current drinking** | | | | | |
| Yes | 136 (16.6) | -2.0 (-5.0 to 1.1) | 1.8 (-1.5 to 5.2) | -3.8 (-8.4 to 0.7) | .53 |
| No | 683 (83.4) | -2.6 (-4.1 to -1.1) | -0.5 (-2.1 to 1.1) | -2.1 (-4.3 to 0.1) | |
| **Obesity** | | | | | |
| Yes | 132 (16.1) | -0.9 (-4.3 to 2.5) | 2.2 (-1.4 to 5.8) | -3.1 (-8.0 to 1.9) | .82 |
| No | 687 (83.9) | -2.8 (-4.3 to -1.4) | -0.6 (-2.2 to 1.0) | -2.2 (-4.4 to -0.1) | |
| **Current hypertension** | | | | | |
| Yes | 181 (22.1) | -3.1 (-5.9 to -0.4) | 1.4 (-1.5 to 4.3) | -4.5 (-8.5 to -0.5) | .08 |
| No | 638 (77.9) | -2.0 (-3.4 to -0.7) | -1.1 (-2.6 to 0.4) | -1.0 (-3.0 to 1.1) | |
| **Current hypercholesterolemia** | | | | | |
| Yes | 696 (85.0) | -2.2 (-3.7 to -0.8) | -0.3 (-1.8 to 1.3) | -1.9 (-4.1 to 0.2) | .58 |
| No | 123 (15.0) | -4.1 (-7.4 to -0.8) | 0.6 (-3.2 to 4.4) | -4.7 (-9.8 to -0.3) | |

© 2025 Zhang PZ et al. *JAMA Network Open*.

The difference of single study outcomes between groups were tested using the mixed-effects model in PROC MIXED, with randomized treatment as a factor and baseline end-point value and clinic center as a covariate using a multiple imputation approach for missing data.

© 2025 Zhang PZ et al. *JAMA Network Open*.

**eTable 5. Changes in the Medication Adherence, Dietary Intake and Physical Activity During the 12-Month Follow-Up**

| Variables | Intervention group (N=410) | Control group (N=409) | Difference between Groups |
|---|---|---|---|
| Medication Adherence | | | |
| Baseline | 6.5 (4.8-7.0) | 6.0 (4.8-7.0) | |
| Month 6 | 6.8 (5.8-8.0) | 6.5 (3.8-8.0) | 0.2 (-0.1 to 0.5) |
| Month 12 | 7.0 (5.8-8.0) | 7.0 (5.0-8.0) | 0.1 (-0.1 to 0.4) |
| Energy intake — kcal/d | | | |
| Baseline | 2868.1±1529.9 | 2780.3±1312.4 | |
| Month 6 | 2306.0±1136.0 | 2313.4±866.9 | -22.0 (-172.9 to 129.0) |
| Month 12 | 2169.0±786.3 | 2293.3±919.0 | -129.4 (-270.8 to 12.1) |
| Carbohydrate intake — % | | | |
| Baseline | 51.2±10.7 | 50.5±10.5 | |
| Month 6 | 49.4±10.2 | 50.2±9.9 | -1.0 (-2.6 to 0.6) |
| Month 12 | 49.3±9.9 | 49.9±9.9 | -0.8 (-2.3 to 0.7) |
| Protein intake — % | | | |
| Baseline | 14.4±3.7 | 14.4±3.6 | |
| Month 6 | 15.0±3.7 | 14.9±3.6 | -0.0 (-0.6 to 0.5) |
| Month 12 | 14.9±3.2 | 15.1±3.8 | -0.2 (-0.7 to 0.3) |
| Fat intake — % | | | |
| Baseline | 35.1±10.4 | 35.7±10.3 | 1.0 (-0.5 to 2.4) |
| Month 6 | 36.3±9.3 | 35.6±9.1 | 0.9 (-0.5 to 2.3) |
| Month 12 | 36.5±13.2 | 35.7±9.0 | |
| Physical activity — METs h/week | | | |
| Baseline | 11.6 (0.8-28) | 11.6 (3.9-28.0) | |
| Month 6 | 15.0 (5.7-29.7) | 15.4 (3.9 -28.9) | -1.3 (-6.9 to 4.2) |
| Month 12 | 11.7 (2.0-24.0) | 11.6 (0-25.6) | -1.7 (-6.8 to 3.4) |
| SF-12 score | | | |
| Physical component summary | | | |
| Baseline | 42.0±5.8 | 41.7±6.8 | |
| Month 6 | 42.2±5.2 | 42.5±5.2 | -0.3 (-1.2 to 0.5) |
| Month 12 | 41.7±5.5 | 41.8±5.9 | -0.1 (-0.9 to 0.7) |
| Mental component summary | | | |
| Baseline | 47.0±5.0 | 47.2±5.5 | |
| Month 6 | 47.2±5.1 | 47.2±4.8 | 0.1 (-0.7 to 0.9) |
| Month 12 | 47.6±4.8 | 47.5±5.4 | 0.1 (-0.7 to 0.9) |

Analyses are conducted with the use of mixed-effects model, with randomized treatment as a factor and baseline end-point value as a covariate using available data from all participants without imputation. METs denotes metabolic equivalents.

© 2025 Zhang PZ et al. *JAMA Network Open*.

**eTable 6. Changes in the Proportions of Medication Recipient at 6 and 12 Months of Follow-Up**

| Variables | Intervention group (N=410) | Control group (N=409) | P value |
|---|---|---|---|
| Baseline | | | |
| Antihyperglycemic agents use | 259 (63.2) | 274 (67.0) | .25 |
| Antihypertensive drugs use | 106 (25.9) | 99 (24.2) | .59 |
| Lipid-lowering agents use | 61 (14.9) | 71 (17.4) | .33 |
| Month 6 | | | |
| Antihyperglycemic agents use | 266 (64.9) | 226 (55.3) | .005 |
| Antihypertensive drugs use | 72 (17.6) | 64 (15.7) | .46 |
| Lipid-lowering agents use | 94 (22.9) | 88 (21.5) | .63 |
| Month 12 | | | |
| Antihyperglycemic agents use | 311 (75.9) | 262 (64.1) | <.001 |
| Antihypertensive drugs use | 90 (22.0) | 74 (18.1) | .17 |
| Lipid-lowering agents use | 104 (25.4) | 91 (22.3) | .30 |

© 2025 Zhang PZ et al. *JAMA Network Open*.

**eFigure 1. Absolute Waist Circumference, Weight, BMI and Diastolic BP Levels According to Randomization Group**

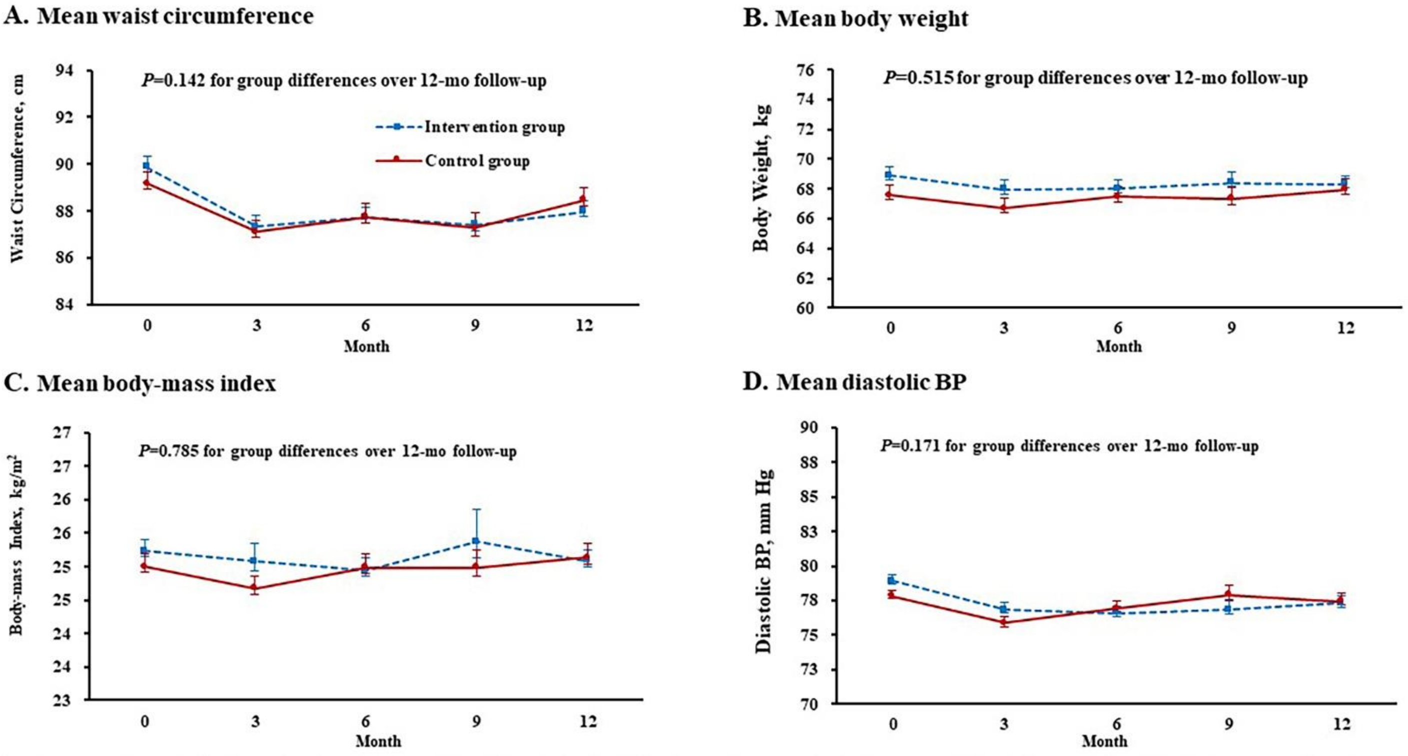


BMI, body-mass index; BP, blood pressure. Error bars represent standard error for the mean. A. Mean waist circumference for both groups over 12 months. B. Mean body weight for both groups over 12 months. C. Mean BMI for both groups over 12 months. D. Mean diastolic BP for both groups over 12 months.

© 2025 Zhang PZ et al. *JAMA Network Open*.

**eFigure 2. Absolute Fasting Glucose, TC, TG and HDL-C Levels According to Randomization Group**

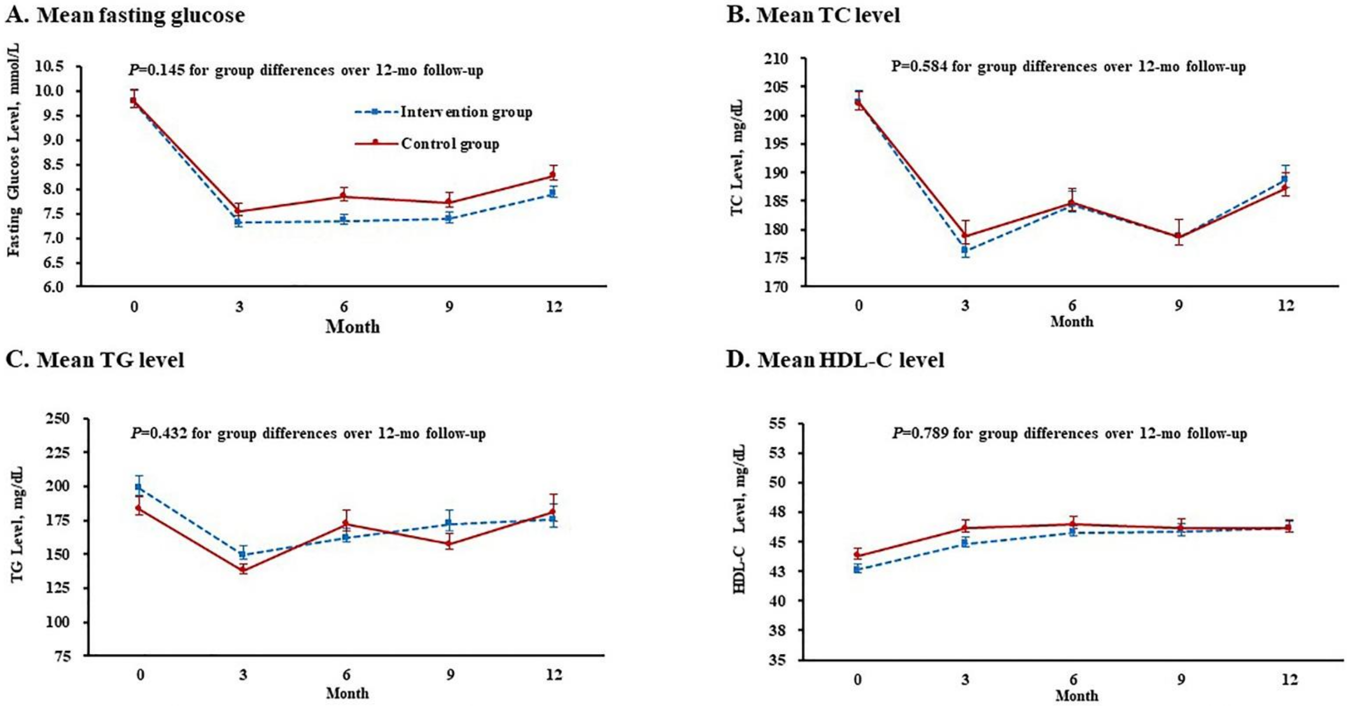


Abbreviations: HDL-C, high-density lipoprotein cholesterol. TC, total cholesterol; TG, triglyceride; Error bars represent standard error for the mean. A. Mean fasting glucose level for both groups over 12 months. B. Mean TC level for both groups over 12 months. C. Mean TG level for both groups over 12 months. D. Mean HDL-C level for both groups over 12 months.

© 2025 Zhang PZ et al. *JAMA Network Open*.
