# Supplementary material for: Mobile App–Based Intervention and Cardiovascular Risk Factors in Patients With Uncontrolled Type 2 Diabetes: A Randomized Clinical Trial
Source: JAMA Netw Open. 2025 Sep 2;8(9):e2529762. doi: 10.1001/jamanetworkopen.2025.29762 (PMC12406060; doi:10.1001/jamanetworkopen.2025.29762)
Supplement: Supplement 3. — Data Sharing Statement [file jamanetwopen-e2529762-s003.pdf]

Data Sharing Statement

Zhang. Mobile App-Based Intervention and Cardiovascular Risk Factors in Patients With Uncontrolled Type 2 Diabetes. *JAMA Netw Open*. Published September 02, 2025. doi:10.1001/jamanetworkopen.2025.29762

Data
Additional Information: name of the trial registry: Text Messaging and Cardiovascular Health in Diabetes Mellitus (TEACH) registry's URL: https://clinicaltrials.gov/study/NCT03724526?cond=NCT03724526&rank=1 trial registration number:ClinicalTrials.gov Identifier: NCT03724526
Data available: Yes
Data types: Deidentified participant data
How to access data: Study data can be shared with qualifying researchers who submit a proposal with a valuable research question as assessed by the study steering committee. A data access agreement may be required. Requests should be directed to Dr. Zhang (e-mail, huijiezhang2005@126.com).
When available: With publication

Supporting Documents
Document types: None

Additional Information
Who can access the data: Study data can be shared with qualifying researchers who submit a proposal with a valuable research question as assessed by the study steering committee. A data access agreement may be required.
Types of analyses: Study data can be shared with qualifying researchers who submit a proposal with a valuable research question as assessed by the study steering committee. A data access agreement may be required.
Mechanisms of data availability: Study data can be shared with qualifying researchers who submit a proposal with a valuable research question as assessed by the study steering committee. A data access agreement may be required.
